# Would manufacturing go for renewable energy? Manufacturers' preference towards sustainability

**DOI:** 10.1016/j.heliyon.2024.e27981

**Published:** 2024-03-16

**Authors:** Siti Noradiah Amar, Mahirah Kamaludin, A.A. Azlina, Muhammad Rias K V Zainuddin, Khairul Izzuddin Sulaiman

**Affiliations:** a Faculty of Business, Economics, and Social Development, Universiti Malaysia Terengganu, Malaysia; b Institute of Tropical Agriculture & Food Security, Universiti Putra Malaysia, Selangor, Malaysia; c Department of Strategic Planning, Sustainable Energy Development Authority (SEDA), Malaysia

**Keywords:** Renewable energy, Choice experiment, Manufacturer, Mixed logit, Willingness to pay

## Abstract

Malaysia needs to fully utilize its renewable energy resources to meet its goal of installed capacity of 31% of renewable energy in 2025 and 40% in 2035. In order to empower renewable energy sources, the government has established a fund known as the renewable energy fund (RE FUND). In Malaysia, most manufacturing sectors contribute to the RE FUND through their monthly electricity bills due to their electricity consumption exceeding 300kwh per month. As Malaysia's highest electricity consumer, the manufacturing sector needs government investment incentives to switch to renewable energy sources to generate electricity. Therefore, this study was conducted to identify attribute preferences of the manufacturing sector due to investing in renewable energy sources. The Choice Experiment method was employed where the Mixed Logit model was chosen to identify the willingness to pay for the manufacturing sectors based on their preferences among the four attributes: types of renewable energy, project location, annual reduction in GHG emissions, and RE FUND. The study results found that the manufacturing sector places the highest value on the project location, where they prefer to improve the project location from current condition to far location. This study can also help to achieve the Goal 7 in the Sustainable Development Goal (SDG), where investment in renewable energy sources can guarantee that all individuals obtain affordable, reliable, sustainable, and modern electricity in 2030.

## 1 Introduction

Renewable energy (RE) sources frequently used to produce electricity include solar photovoltaic, biomass, biogas, and small hydropower. Natural resources, including sunshine, plants, air, wind, and animal waste, are the primary sources of renewable energy where most are completely free. Malaysia's renewable energy resources must be fully utilized to enable the government to use installed capacity renewable energy sources at a rate of 31% in 2025 and 40% in 2035. Malaysia's RE share objective is well-aligned with its pledge to decrease its economy-wide carbon intensity (relative to GDP) by 45% in 2030 as part of its global climate commitment. At present, only 23% of renewable energy sources have been implemented in Malaysia [1]. The Malaysia Renewable Energy Roadmap (MyRER) was designed with the utmost confidence to expedite decarbonization in the electricity sector, surpassing the 2035 milestone. Malaysia is among the major oil and gas producers, but its domestic production cannot satisfy its demand for petroleum and related products. To meet its energy needs and support various industries that depend on these resources, Malaysia must import crude oil, refined petroleum products, and other petrochemicals. It was predicted that the nation would continue to rely more on imported oil [2].

Tenaga Nasional Berhad (TNB) is Malaysia's biggest electricity utility company. As a state-owned entity, it produces, transmits, and distributes electricity across Peninsular Malaysia and Sabah. TNB categorizes electricity users into two types: residential and business. The manufacturing sectors fall under the business category. Residential or domestic consumers refer to individuals residing in private homes not intended for commercial purposes, such as hotels or boarding houses. These consumers typically refrain from engaging in any business, trade, or professional work. In contrast, energy is vital in manufacturing industries for producing various goods. The monthly minimum electricity bill for residential users is RM3.00, and RM7.20 for businesses in the manufacturing sector. The manufacturing sector has four tariff types, including (i) low voltage tariff, (ii) medium voltage general tariff, (iii) medium voltage peak/off tariff, and (iv) high voltage peak/off tariff, as opposed to residential users with only one tariff type [3].

In Malaysia, the manufacturing sector consumes the most electricity energy among other users [4], mainly generated from fossil fuel sources, which emit exhaust gases that contribute to the greenhouse effect and can raise the temperature of the Earth's atmosphere. The continuous and depending on use of fuel resources to generate electricity could accelerate global warming. The community needs to be aware of the benefits of renewable energy sources, especially in the manufacturing sector. However, the maintenance cost of renewable energy sources is the main factor behind these resources not being fully implemented. It cannot be denied that renewable energy is more expensive than the most cost-effective fossil-fuel option and costs three times more than surplus electricity [5]. While renewable energy sources can be expensive, there are ways to reduce the overall costs and balance the marginal costs to make them more financially feasible [6].

The main objective of this study is to evaluate the economic values of renewable energy (environmental goods) and examine how we can place a monetary value on environmental goods using economic valuation methods. The specific objective of this study aimed to determine the manufacturing sector's willingness to pay (WTP) for investment in the renewable energy sources in their production. This study is the first to conduct a Choice Experiment (CE) in Malaysia to investigate the willingness to pay (WTP) for renewable energy among manufacturers. While previous studies have mainly focused on consumers' and households' preferences for renewable energy. Specifically, this study contributes to the literature by using a CE method and quantifying manufacturer preferences for renewable energy investments in Malaysia. From a scientific perspective, the manufacturing industry is a significant consumer of electricity, and as such, the findings drawn from this study will likely serve as a stimulus for increased investment in sustainable energy solutions. This is a crucial step towards mitigating the greenhouse gas emissions that are generated by this sector. This paper is organized starting from the introduction section, which introduces the importance of renewable energy sources to replace fossil fuel energy sources. The literature review section highlights the economic value of renewable energy and previous studies on the WTP towards energy sources. Meanwhile, the methodology section describes the method and data collection involved in the study. Next, the result section demonstrates the analysis of the CE to estimate the marginal willingness to pay, and the last section summarizes and concludes the study.

## 2 Literature review

Renewable energy (RE) sources provide multiple benefits and values compared to conventional fossil fuels, where RE is a reliable and enduring energy choice that can replenish naturally over time. In contrast to finite resources such as fossil fuels, sustainable energy sources offer a dependable and long-lasting solution. Besides, depending on the renewable energy sources decreases our reliance on imported fossil fuels, enhancing energy security and decreasing susceptibility to price fluctuations and geopolitical tensions.

Table 1 describes the economic value obtained from the renewable energy sources. The economic value of renewable energy sources is viewed from many perspectives and includes numerous direct and indirect values for both people and the environment. Direct use value includes using renewable energy sources either consumptively or non-consumptively. Consumptive use involves taking the renewable energy sources for consumptive/use purposes. Several renewable energy sources in Malaysia are still underutilized, including solar, biomass, biogas, and small hydropower.

**Table 1:** The value obtained from renewable energy sources.

The value obtained from renewable energy (RE) sources					
Value	Use value – benefits obtained from physical use or access to environmental goods.				Non-use value – Benefits obtained from the existence of the resource.
	Direct use value – Goods or services that can be used directly.		Indirect use value – Benefits enjoyed indirectly.	Optional value – The amount that the factory is willing to pay to avoid future destruction of the resource.	Non–use – Existence value or value left for future generations.
	Consumptive (Extractive)	Not consumptive (Not extractive)			
Examples	- Solar - Biomass -Biogas -Hydropower -Wind	-Research and development -Education -Tourism, i.e., from a visit to the hydro dam	-Reduce coal imports from Indonesia. -Maintain a clean environment. -Reduce air and noise pollution -Mitigate the negative impacts of global warming.		Existence value – Value from knowledge about renewable energy. Bequest value – Value from the awareness that future generations will benefit from the RE sources.
Examples Of Economic Value	The transition to a green economy needs an investment of RM637 billion [7]				
	The South Korean public is WTP USD0.38 for raising the ratio of renewable energy through an increase in the monthly electricity bill [8]. The WTP for RE to expand in the generation mix would result in solar photovoltaic and wind power's share of generation increasing from 0.7% to 4.3% by 2050 in Japan [9]. Turkish residents were willing to spend 9.25 TL/month for a 20% share in renewable energy production and 4.77 TL for a 30% share in total energy production [10].		Households are WTP USD0.0088 for renewable energy investment to decrease air pollution [11]. On average, people are willing to pay PLN 83.7 (approx. USD 21.47) per month to support using renewable energy and reduce environmental pollution in Krakow, Poland [12]. Respondents are, on average, willing to pay, in addition to their electricity bill, £29.65 (s.e5.50) to decrease GHG emissions by 1% a year [13]		

Note: Based on author's compilations.

Numerous studies have investigated energy consumption trends in the manufacturing sector across various countries. Notable researchers, including Malinauskaite et al. [14], Brodny and Tutak [15], and Wang et al. [16], have conducted such studies based on statistical data. However, the majority of these studies have centered on secondary data, rather than primary data. It should be noted that primary data collection offers various benefits compared to other methods. As data is collected directly from the source, primary data is typically more reliable and precise. Research on renewable energy in Malaysia is of utmost importance, given its close link with the objectives of the Malaysia Renewable Energy Roadmap (MyRER). The MyRER seeks to raise the proportion of renewable energy to 31% or 12.9 GW by 2025, and 40% or 18.0 GW by 2035 [17]. This endeavor is anticipated to aid Malaysia's pledge to cut down greenhouse gas (GHG) emissions as per the Paris Agreement, led by the United Nations Framework Convention on Climate Change (UNFCCC).

This section reviews previous studies on renewable energy sources utilizing primary data by employing the choice experiment (CE) method. However, it is worth noting that only a few studies have employed this method for renewable energy sources, focusing on those using the choice experiment method. Table 2 depicts several studies that have been conducted in developed countries such as Scotland [18], Sweden [19], England [13], Korea [11], European England [20], US and Japan [21], South Korea [22], West Virginia [23], and Poland [24]. However, there are also an increasing number of, but very limited, studies that have been conducted in other developing countries such as Indonesia [25], Africa [26], and Kenya [27].

**Table 2:** Studies Related to Willingness to Pay (WTP) for the attributes of renewable energy investment in developed countries.

No.	Author	Survey Area	Attributes	Signs	Value of WTP
1.	[18]	Scotland	Landscape impact	+	8.10
			Wildlife impact	+	4.24
			**Air pollution impact**	**+**	**14.13**^1^
			Employment impact	Not significant	0.32
			Price of electricity	–	
2.	[19]	Sweden	Reduction of noise level	+	0.67
			**Location**	**+**	**3.47**
			The height of the tower of the turbine	+	0.26
			Grouping	–	1.64
			Price	–	
3.	[13]	England	**Annual reduction in GHG emissions**	**+**	**29.65**
			Annual length of electricity shortages	–	0.36
			Changes in the number of employees	+	0.02
			Electricity bills	–	
4.	[11]	Korea	Landscape impact	Not significant	2.52
			Wildlife impact	+	6.85
			Air pollution impact	+	8.40
			**Employment impact**	**+**	**10.87**
			Price of electricity	–	
5.	[20]	European Union (England, Wales, Scotland)	**Type of system** Capital cost Annual energy saving Maintenance cost	**+** – + –	**3.0341** −1.1122 0.2957 −0.0922
6.	[21]	US and Japan	Monthly bill Air emission **Fuel mix**	**-** **-** **-**	0.34 (US) 0.26 (Japan) **0.74 (US) 0.31(Japan)**
7.	[22]	South Korea	**Installation location** Installation scale Operation Electricity use Price	**+** **+** **+** **+** **-**	**4286** 3712 2885 3731
8.	[23]	West Virginia	Energy source Proximity Additional monthly fees	+ –	21.79
9.	[24]	Poland	**Minimum distance to residential areas** Size of renewable energy production sites Number of renewable energy production sites Share of landscape not used for renewable energy expansion High-voltage transmission lines Monthly surcharge or rebate to energy bill	**+** Not significant Not significant + + –	**5.66** 0.93 0.09 9.15 2.64

1 Attributes in bold are the highest preferences that respondents are willing to pay.

**Fig. 1:**
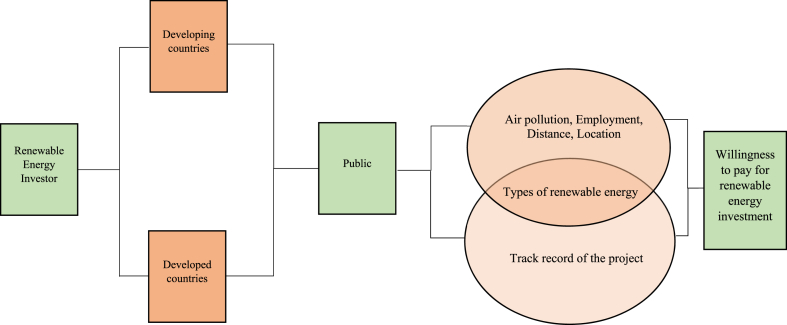
Determinants affecting the public's willingness to pay for renewable energy sources in developed and developing countries.

Various factors might influence the willingness of the public to pay for investments in renewable energy sources in both developed and developing countries (Fig. 1). In developed countries, households place a high value on five attributes: types of renewable energy, air pollution reduction, employment, distance, and location. Meanwhile, in developing countries, the highest value that households place is on the types of renewable energy and the track record of project developers. Both developing and developed countries demonstrate types of renewable energy as one of the attributes that they are willing to pay with high value. In this case, the types of renewable energy play an essential role for both countries.

Most previous studies focused on the willingness to pay for renewable energy investment based on the household perspective without directly investigating the industrial and commercial perspectives [25,28], particularly in developing countries using the choice experiment method. There are only a few studies that investigate renewable energy investment from manufacturer perspectives without assigning the value of WTP but by stating which one of the attributes is critical to invest in Refs. [[29], [30], [31]] Therefore, it is crucial to close the gaps by investigating the willingness to pay for renewable energy investment from the manufacturer's perspective, for example, by assessing the value of each attribute based on their preferences.

## 3 Research methodology

### 3.1 Choice experiment (CE) method

Contingent Valuation Method (CVM) and Choice Experiment (CE) Method are often used to determine the benefits of a development activity compared to the loss of natural resources and services in monetary term. Both methods have their roots in the Stated Preference approach. A survey will be conducted among the selected respondents in order to directly obtain the monetary values or indirectly to assess the willingness of the respondents to cover the cost (willingness to pay) of economic losses if the development activity is cancelled. The amount is considered as an estimation of the monetary value for the environmental component.

In contrast to CVM, which contrasts the advantages of development with the natural world, CE frequently compares several development options that have varying effects on the environment as well as varying benefits to users. Even though these approaches are perceived to have numerous flaws and drawbacks, CVM in particular is frequently used, particularly to calculate compensation when harm is caused by economic and development activities.

Studies related to renewable energy using CE methods in Malaysia are still limited in number. Most previous studies on renewable energy employed the CVM [[32], [33], [34]]. The CVM has become the most common non-market valuation method used to estimate the benefits of environmental goods and services [35]. However, there are some doubts, especially in situations with multiple options and several attributes [36]. The CE is another alternative to nonmarket valuation techniques of the Stated Preferences method that allows everyone to make the best choice among the other options. Researchers have a favorable view of the choice experiment method to evaluate the benefits of non–market environmental goods or services [[37], [38], [39], [40]]. Therefore, implementing this study increases the number of research highlights related to renewable energy sources through the CE method.

This study was conducted among the manufacturing sectors in Johor, a state growing rapidly and using the most energy in Malaysia. There are 8046 manufacturing sectors in various sub-sectors in Johor, the second highest after Selangor [41]. The seven sub-sectors include electrical, petroleum, timber, food and beverages, textiles, minerals, and transportation. Johor has ten districts: Tangkak, Segamat, Kluang, Mersing, Kota Tinggi, Kulai, Johor Baharu, Pontian, Batu Pahat, and Muar.

**Fig. 2:**
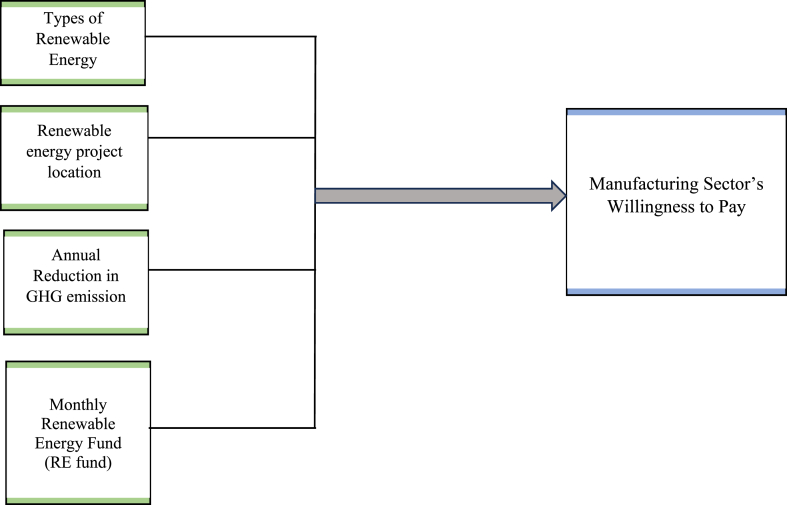
Conceptual framework.

Fig. 2 presents the conceptual framework that will be used in this study. According to Johnson et al. [42], 70% of existing choice experiment study use from three to seven attributes. As for this study, we will use four attributes to identify the willingness to pay of renewable energy among the manufacturing sectors in Johor. The four attributes that will be used in this study are types of renewable energy, renewable energy project location, annual reduction in GHG emission, and monthly renewable energy fund (RE Fund). Willingness to pay used in this study refer to the maximum price that manufacturing sectors are willing to pay to show their preferences in renewable energy service.

**Fig. 3:**
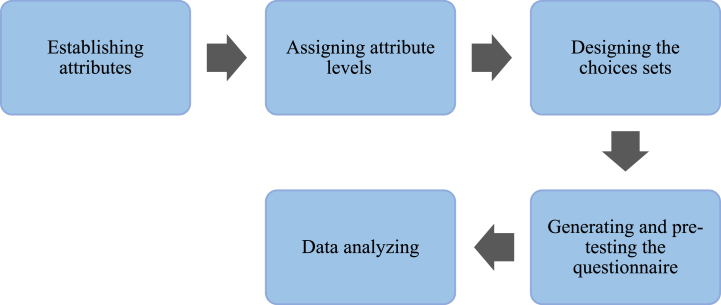
Steps of Choice Experiment (CE) method.

Several steps must be followed to implement the choice experiment method successfully. The first critical step is identifying the attributes relevant to the study's objectives. Once these attributes have been identified, the second step is to assign a level to each selected attribute. The third step is to design sets of options that will produce alternatives for respondents. The fourth step involves creating a pre-test questionnaire using the sets of choices built. Lastly, the fifth step is to analyze the data using the Multinomial Logit and Mixed Logit models, providing a clear understanding of the results. Fig. 3 shows the steps included to conduct choice experiment method.

The use of the CE method requires the identification of attributes. This study considers four attributes: (i) the types of renewable energy sources (TORE), (ii) project location (PL), (iii) annual reduction of GHG emission (GHG), and (iv) Renewable energy fund (RE Fund). Table 3 exhibits a comprehensive list of attributes and their respective descriptions and levels.

**Table 3:** Attributes, attributes level, and descriptions.

Attributes	Level	Descriptions
Types of renewable energy (TORE)	Solar photovoltaic	Preference for solar photovoltaic generation to support the government target to achieve 40% of renewable energy installed capacity by 2035 under the FiT.
	Biomass	Preference of biomass generation to support the government target to achieve 40% of renewable energy installed capacity by 2035 under the FiT.
	Biogas	Preference of biogas generation to support the government target to achieve 40% of renewable energy installed capacity by 2035 under the FiT.
	Small hydropower	Preference for small hydropower generation to support the government target to achieve 40% of renewable energy installed capacity by 2035 under the FiT.
Project Location (PL)	Unchanged	Renewable energy project plants remain located in Pasir Gudang and Pengerang.
	Near	Renewable energy project plants are close to the factories.
	Far	Renewable energy project plants are far from the factories.
Annual reduction of GHG emission (GHG)	29.4%	Reduction of GHG emissions in 2005.
	36.69%	Average reduction in GHG emissions from 2005 to 2030.
	45.0%	The government's target is to reduce carbon emissions by 45% in 2030.
Monthly Renewable Energy Fund (RE FUND)	1.6%	Maintain the current renewable energy fund charged to manufacturing sectors.
	2.2%	Increase by 0.6% from the current charge.
	2.8%	Increase by 1.2% from the current charge.

The selection of the first attribute, the types of renewable energy sources (TORE), was chosen based on the government's objective to achieve 40% of the installed renewable energy capacity by 2035 [43]. The highest amount of renewable energy sources used was from solar energy (424, 191 MW), followed by biomass (228, 540 MW), biogas (216, 890 MW), and small hydropower (75, 552 MW) [43]. The government has been looking to promote using renewable energy sources other than solar to meet its target. This is because the current use of renewable energy sources has not yet reached the desired level. As part of this initiative, the government has introduced the Feed-in-Tariff (FiT) program to encourage the use of biomass, biogas, and small hydropower.

The second attribute is the project location (PL), which demonstrates the distance between the location of the renewable energy power plant project and the factory area. The attribute is based on the existence of the renewable energy power plants in several areas in Malaysia, including Johor. The distance between the location of the renewable energy power plant project and the factory area is crucial to ensure that the activity of generating electricity from renewable energy will not interfere with manufacturing activities. Johor currently has three renewable energy power plants, one in Pengerang and two in Pasir Gudang [44].

The third attribute is the annual reduction of greenhouse gas emissions (GHG). The attribute aligns with the government's objective, which aims to reduce carbon emissions by 45% in 2030. In 2005, Malaysia successfully recorded a reduction in GHG emissions by 29.4% (12th Malaysia Plan 2021–2025) [45]. To achieve a GHG reduction of 45% by 2030, this study used the Compound Annual Growth Rate (CAGR) calculation method as used by Waldau et al. [46]. This study used the average value of GHG reduction to avoid a large difference between level 1 (29.4%) and level 3 (45.0%).

The last attribute is the monetary attribute, which is the monthly renewable energy (RE Fund). The RE Fund is an additional charge for all electricity users who use electricity above RM77 or 300 kWh per month registered with the electricity utility company TNB. The current charge for the RE Fund is 1.6% of each consumer's electricity bill. The attribute level is based on the difference in the percentage increase of the RE Fund imposed by the government on electricity consumers from 2011 until 2014, which is 0.6%. According to the Chief Executive Officer of SEDA Malaysia, Dato’ Hamzah bin Hussin, this value of 0.6% is the optimal value to achieve the use of renewable energy sources targets in 2025 and 2035, while the investment value that does not exceed 3% is reasonable and that does not burden the manufacturing sectors (H. Hussin, personal communication, June 8, 2021).

### 3.2 Mixed Logit (ML) model

In this study, the Mixed Logit (ML) model was applied to determine the manufacturing sectors' willingness to pay to invest in renewable energy sources. A linear function of an attribute vector (X_1_, X_2_, X_3_, X_4_) = (Types of renewable energy, project location, annual reduction of GHG emission, Renewable Energy fund) was used to illustrate the exception of the error factor in the utility function model. Equation (1) shows the parameters to be estimated for each attribute that influences the respondent's utility.

(1) U = *β*_*1*_*X*_*1*_ + *β*_*2*_*X*_*2*_ + *β*_*3*_*X*_*3*_ + *β*_*4*_*X*_*4*_ + ε

The CE involves calculating marginal willingness to pay (MWTP) for each attribute as in Equation (2):

(2) $${\text{MWTP}}_{j}=-\;\frac{\text{Vj}}{\text{Vp}}$$

Where the MWTP is a marginal willingness to pay for an attribute *j*, *V*_*j*_ is the value (mean coefficient) of the attribute *j,* and *V*_*p*_ is the value (mean coefficient) of the price (monetary) attribute.

### 3.3 Data sampling

The study was carried in Johor, Malaysia, specifically in the manufacturing sector. Johor is the fastest-growing state that consumes Malaysia's most energy. There are 8046 manufacturing sectors in various sub-sectors in Johor, the second highest after Selangor [41]. The sub-sectors include electrical, petroleum, timber, food and beverages (F&B), textiles, minerals, and transportation. Johor consists of ten districts: Tangkak, Segamat, Kluang, Mersing, Kota Tinggi, Kulai, Johor Baharu, Pontian, Batu Pahat, and Muar.

Sampling techniques used in choice experiment methods are simple random sampling and stratified sampling [47]. The survey was conducted exclusively with manufacturers holding the position of managing directors or general managers, employing purposive sampling based on a non-probability sample. Face-to-face interviews were performed to obtain respondent information and collect data. In this study, 30 individuals working in the manufacturing sectors were selected to participate in a pre-test and evaluate the questionnaire. The data collected from these 30 respondents were not merged in the final data set. The pre-test had to be conducted twice due to inconsistent attributes and attribute level selection in the first round of the pre-test. For this study, we utilized the sample size calculation method suggested by Ref. [48]. The sample size calculation method was identical to the one employed by Bridges et al. [49]. Equation (3) provides a reliable calculation for the appropriate sample size in the CE method.

(3) *N* ≥ *500c / (t x a)*

Where “c” is the largest number of levels for any attribute, “a” is the number of alternatives on the choice card (excluding the status quo alternatives), “t” is the number of choice cards given to each respondent, and “N” is the total number of respondents. Thus, this study applied the calculation of the data sampling as below;

(4) $$\begin{array}{c} N\;>\;500\left(4\right)\;/\;\left(8\;x\;2\right)\\ N\;>\;2000\;/\;16\\ N\;>\;125 \end{array}$$

In order for this study to adequately portray the manufacturing sector in Johor, it was required to collect data from a total of 125 different organizations, according to calculation in Equation (4). This sample was deemed appropriate and reliable for our study.

This study employs a primary data collection method through the distribution of questionnaires to respondents. Prior to the interview session, a consent form was presented to each respondent to secure their permission to participate in the interview. Only those who were granted such permission were allowed to answer the questionnaire, thus ensuring the accuracy and reliability of the data collected. Besides, the consent form serves as a crucial document that provides respondents with a detailed and comprehensive explanation of the research survey, including its purpose, objectives, and the type of data being collected. This ensures that respondents are fully informed and have a clear understanding of the research objectives before agreeing to participate. In this case, the consent form thus plays a vital role in ensuring that ethical considerations are met in the research process, and respondents' right to informed consent is upheld.

## 4 Results

The results of the study are divided into three sub-sections, including (i) descriptive analysis, (ii) knowledge and perception of manufacturers, and (iii) Mixed Logit analysis (ML) results.

### 4.1 Descriptive analysis

This study was conducted among various manufacturing sub-sectors in Johor, Malaysia. Face-to-face interviews were carried out with managing directors or general managers responsible for providing information on electricity generation in their factories. Two enumerators successfully conducted interviews with 200 manufacturing sectors in Johor in an efficient manner. A purposive sampling technique was employed to gather data over a span of four months, specifically from April 2022 to August 2022. The questionnaires were distributed to factories of various sub-sectors within Johor districts, with different distributions in each district (Table 4), as each district has different manufacturing sectors and sub-sectors.

**Table 4:** Distribution of questionnaire according to district and sub-sector, n = 200.

Subsectors Districts	Electrical and electronics products	Petroleum, chemical, rubber, plastic products	Woods products, furniture, paper products, printing	Food, Beverages, & Tobacco	Textiles, Wearing apparel, Leather products & footwear	Non–metallic mineral products, basic metal, fabricated metal products	Transport equipment and other manufacturing	Total (n)
Kluang	1	8	1	3	5	–	10	28
Mersing	–	–	–	–	1	–	–	1
Kota Tinggi	–	5	2	2	3	1	1	14
Kulai	2	2	–	–	–	1	1	6
Johor Baharu	9	8	6	7	3	10	13	56
Pontian	–	–	–	–	–	–	1	1
Batu Pahat	8	8	12	13	11	7	10	69
Muar	2	1	3	1	1	10	2	20
Tangkak	–	–	1	–	–	1	2	4
Segamat	1	–	–	–	–	–	–	1
**Total (n)**	23	32	25	26	24	30	40	200

### 4.2 Knowledge and perception of manufacturers

Table 5 shows the knowledge and perception of manufacturers towards renewable energy sources. Out of 200 respondents interviewed, 76% strongly agreed that maintaining renewable energy resources is too expensive, while 20.5% agreed, 2.5% were undecided, and 1% disagreed. This clearly shows that the high maintenance costs might be the reason for Malaysia's underutilized renewable energy resources. The second item concerns the importance of using renewable energy sources for future generations to enjoy a clean environment. Out of 200 respondents, 75.5% strongly agreed that using renewable energy is essential for future generations to enjoy a clean environment. Meanwhile, 21% agreed, 3% were undecided, and 0.5% disagreed with this statement. This shows that most of them are aware of the responsibility of taking care of the environment through renewable energy sources to ensure that future generations can enjoy a clean environment. The third item was related to the granting of subsidies by the government for renewable energy sources. Out of 200 respondents, 78.5% strongly agreed that the government should provide subsidies for renewable energy sources. Meanwhile, 18.5% of the respondents agreed, 3% undecided, and 0.5% disagreed. This shows that the majority of the manufacturing sectors strongly agreed that the government should subsidize the renewable energy sources.

**Table 5:** Knowledge and perception of manufacturers towards renewable energy.

Statements	1 Strongly disagree	2 Disagree	3 Undecided	4 Agree	5 Strongly agree
i. Renewable energy resources are underutilized since sustaining them is too expensive.	0.00	1.00	2.50	20.50	76.00
ii. Adopting renewable energy sources is crucial so future generations can enjoy a clean environment.	0.00	0.50	3.00	21.00	75.50
iii. The government should fund renewable energy sources with subsidies.	0.00	0.50	3.00	18.50	78.50

### 4.3 Mixed Logit results

This section presents the results of the study using the ML model. The results of this study are divided into three parts, namely the results of the study for the simple ML model, the ML model with interactions, and the calculation of marginal willingness to pay.

#### 4.3.1 The simple Mixed Logit (ML) model

This section shows Mixed Logit (ML) incorporating levels model specification for the renewable energy investment of their attributes. The model specification for ML incorporating levels is as follows:

U = *β*_*1*_*X*_*TORE2*_ + *β*_*2*_*X*_*TORE3*_ + *β*_*3*_*X*_*TORE4*_ + *β*_*4*_*X*_*PL2*_ + *β*_*5*_*X*_*PL3*_ + *β*_*6*_*X*_*GHG2*_ + *β*_*7*_*X*_*GHG3*_ + *β*_*8*_*X*_*RE FUND*_ + ε

The results of the ML model are shown in Table 6.

**Table 6:** Regression of Mixed Logit model.

Variables	Coefficient (β)	Std. Error	t-value
TORE2	−0.0782	0.0995	−0.79
TORE3	−0.1445	0.1143	−1.27
TORE4	−0.0778	0.1050	−0.74
PL2	0.2730***	0.0843	3.24
PL3	0.3488***	0.0801	4.35
GHG2	0.1272	0.1008	1.26
GHG3	0.0697	0.0935	0.74
RE FUND	−0.1224*	0.0714	−1.71
*Summary Statistics*			
Number of observations 1600			
Log Likelihood −1716.0239			
Log Likelihood, No coefficients −1729.6114			
Pseudo R^2^ 0.0079			
Adjusted Pseudo R^2^ 0.0022			

Note: (*) 10% level, (**) 5% level, (***) 1% level.

The coefficients of TORE2, TORE3, and TORE4 were negative in their relationship and insignificant (Table 6). The negative signs imply that respondents are not willing to pay for renewable energy and prefer to stay in the current condition (status quo) for electricity generation in their factories. The coefficients of PL2 and PL3 were positive in their relationship and significant at a 1% level. These positive relationships indicate that respondents moved from the current PL (status quo) to a higher level (PL2 and PL3). The GHG2 and GHG3 were also positive in their relationship but not significant. The variable with a positive sign implies that respondents need a higher reduction of GHG emissions per year rather than the status quo (level 1). Respondents, however, did not prioritize these attributes in their renewable energy preferences. The RE FUND variable had a negative sign and was statistically significant at a 10% level. It confirms our prior expectation that as the proposed RE Fund increases, manufacturers want to contribute less because of decreased utility levels.

#### 4.3.2 The Mixed Logit (ML) model with interaction

The ML model can be more precise by improving the model itself. Many alternatives can be applied to improve the model fit in the CE. One of them is by including the respondent's socio demographic offers heterogeneity in choices [50,51]. Though, this study added respondent's information characteristics as below.

(i) Electricity generation from renewable energy (RE)
(ii) Types of renewable energy used (TOR)
(iii) Program involved (PRO)
(iv) Types of subsector production (SUB)
(v) Kumpulan Wang Tenaga Boleh Baharu (KWTBB) – RE Fund in Malaysia

By including new attributes, it slightly improved the model fit compared to the ML basic model. The insertion of respondents’ background attributes is an essential step for accuracy in estimating the model [52]. Table 7 shows the results of the ML logit for the interactions model. The ten variables included as attribute interactions in the model influence the model fit as below:

U = β_1_X_TORE2_ + β_2_X_TORE3_ + β_3_X_TORE4_ + β_4_X_PL2_ + β_5_X_PL3_ + β_6_X_GHG2_ + β_7_X_GHG3_ + β_8_X_RE FUND_ + β_9_X_TORE4_Y_RE_ + β_10_X_TORE4_Y_TOR_ + β_11_X_TORE2_Y_SUB_+ β_12_X_TORE3_Y_SUB_ + β_13_X_TORE4_Y_SUB_ + β_14_X_TORE2_Y_KWT_ + β_15_X_TORE3_Y_KWT_ + β_16_X_PL2_Y_KWT_ + β_17_X_PL3_Y_KWT_ + β_18_X_GHG3_Y_KWT_ + ε

**Table 7:** Best ML model with interactions.

Variables	Coeff	t-value
TORE2	1.2714***	4.08
TORE3	−1.6263***	−3.57
TORE4	0.0930	0.46
PL2	1.7695***	6.05
PL3	3.1312***	6.86
GHG2	0.2323***	2.72
GHG3	1.1420***	4.95
RE FUND	−0.1885***	−2.63
TORE4_RE	−1.4523**	−2.50
TORE4_TOR	0.7142***	3.42
TORE2_SUB	−0.1379***	−3.46
TORE3_SUB	−0.1274***	−3.10
TORE4_SUB	−0.1047***	−2.58
TORE2_KWT	−0.8488***	−3.04
TORE3_KWT	1.7973***	4.19
PL2_KWT	−1.5674***	−5.27
PL3_KWT	−2.8368***	−6.25
GHG3_KWT	−0.9540***	−4.02
*Summary Statistics*		
No. of Observations	1600	
Log-likelihood	−1669.1282	
Pseudo R2	0.0350	
Adjusted Pseudo R2	0.0268	

Note: (*) 10% level, (**) 5% level, (***) 1% level.

Based on Table 7, the sign of TORE was positive at level two and was significant at the 1% level. This implies that manufacturing sectors prefer ‘biomass’ rather than ‘solar’ (SQ) as renewable energy services. However, when we proposed to level three, they rejected it. It proves that respondents still choose the current situation. The main attributes of PL and GHG were positive at levels two and three at 1%, implying that manufacturing sectors prefer to move from the current condition.

RE variable is the variable that demonstrates whether the manufacturing sectors generate electricity from renewable energy or not. The interactions produced a negative sign to the variable of TORE4_RE significant at a 5% level. This presents that respondent who have used renewable energy sources to generate electricity in their respective factories do not associate using renewable energy sources with the type of renewable energy sources used to generate electricity. This group of respondents are aware of the importance of using renewable energy sources to generate electricity in their factories, but they do not consider the type of renewable energy sources used to generate electricity in their factories.

The results of this study are surprising when the interaction between the variables TORE4 and types of renewable energy used (TOR) was significant and illustrated a positive relationship (TORE4_TOR). Manufacturing sectors that used solar photovoltaic to generate electricity in their factories are concerned about the types of renewable energy used, as shown by a significant level at 1% level. This group of respondents certainly believed that the types of renewable energy used besides solar will also help the government achieve its goal of using renewable energy sources by 31% in 2030.

The type of subsector production (SUB) was significant and illustrated a negative relationship in these variables, such as TORE2_SUB, TORE3_SUB, and TORE4_SUB. It implies that some manufacturing sectors do not relate the type of subsector production (SUB) with any TORE level. They are probably choosing the current situation in renewable energy services. The variable of TORE2_KWT showed a negative sign with a 1% significant level. It shows that some manufacturing sectors do not relate KWTBB (RE Fund) with any TORE level. They are probably choosing the current situation in energy consumption. Conversely, manufacturing sectors that are aware about KWTBB take the types of renewable energy very seriously as indicated by a positive sign in variables of TORE3_KWT, which was significant at a 1% level. The variables of PL2_KWT and PL3_KWT both showed a negative relation with 1% significant level. This implies that respondents reject the proposal and prefer the current situation of energy consumption. The variable of GHG3_KWT showed a negative relationship with a 1% significant level. This group of respondents agreed that the current situation of energy consumption is better than the proposed level. They did not relate the KWTBB (RE Fund) with any level of reduction of GHG emission. The explanatory power for the interaction ML model was higher than for simple ML model. The adjusted pseudo R2 in the simple ML model was 0.0079, while in the interaction ML model, it was 0.0350. This indicates that the inclusion of respondent's information characteristics improves model fit.

#### 4.3.3 Marginal willingness to pay

Both models of the basic ML model and interaction ML model can be used for further estimation of the Marginal Rate of Substitution (MRS) between different choice attributes. This part illustrates the differences in marginal values in all attribute levels. The marginal values are calculated by the coefficient differences between the two attribute levels. In addition, the arrows in the table illustrate the directions of change in attribute levels. Table 8 contains two models: simple ML model and interaction ML models for estimated marginal values of difference in attribute levels.

**Table 8:** Estimation of the marginal willingness to pay (MWTP).

Attribute Levels	ML Model (%)		Average (%)
	Simple	Interaction	
TORE1→TORE2 *Solar photovoltaic to Biomass*	−0.64	6.75	3.06
TORE2→TORE3 *Biomass to Biogas*	−1.18	−8.63	−4.91
TORE3→TORE4 *Biogas to Small hydropower*	−0.64	0.49	−0.08
PL1→PL2 *Unchanged to Near*	2.23	9.39	5.81
PL2→PL3 *Near to Far*	2.85	16.61	9.73
GHG1→GHG2 *29.4% to 36.7%*	1.03	1.23	1.13
GHG2→GHG3 *36.7% to 45.0%*	0.57	6.06	3.32

The results of the MWTP estimates are shown in Table 8. On average, the respondents were willing to pay as much as 3.06% per month for types of renewable energy sources (TORE) from “solar photovoltaic to biomass.” The result illustrates that the respondents are satisfied when types of renewable energy are improved at the “solar photovoltaic to biomass” level and are not expecting more than that in services. This is based on the negative value at the level of “biomass to biogas” and “biogas to small hydropower”. Next, on average, respondents were willing to contribute as much as 5.81% per month to improve renewable energy project location (PL) from “unchanged to near” and 9.73% for “near to far”. This implies that respondents are willing to pay more for far renewable project locations from their factories. Meanwhile, in terms of annual reduction GHG emission (GHG), the respondents were willing to pay as much as 1.13% per month to improve annual reduction from 29.4% to 36.7% and 3.32% per month for 36.7%–45.0%. This simply means that the respondents support reducing annual GHG emissions from “36.7%–45.0%” rather than “29.4%–36.7%”. This is based on the average value that is three times higher in “45.0%” than “36.7%”.

## 5 Discussion

According to the findings of this study, the manufacturing sector places considerable economic value on the project location attribute. As a result, these sectors tend to relocate the existing renewable energy location to a more distant area from their manufacturing location. The findings are consistent with the study conducted by Bartzack et al. [24] in Poland, where respondents placed a high value on the attribute of distance between the project location and the respondent's residential area. This shows that the ‘project location’ attribute is an important attribute that needs to be included in the study because it reflects the respondent's preferences to invest in renewable energy. Moreover, the location of the project also plays a crucial role as it helps avoid any disruptions that could hinder manufacturing activities and their production. Manufacturers may consider the environmental impact of their operations. In addition, to mitigate environmental impact, renewable energy plants should be located away from manufacturing facilities to protect local ecosystems and minimize visual and environmental impacts.

This study differs from previous studies conducted in developing and developed countries (Fig. 1), as this study does not focus on ‘types of renewable energy’ attribute. The study reveals that the manufacturing sector in Johor, Malaysia, is reluctant to adopt alternative renewable energy sources other than solar energy to generate electricity in their factories. The manufacturing sector is not willing to invest with a higher value for the type of renewable energy sources and prefers to be in the current condition (status quo), which is to consume solar energy. The majority of the participants were not acquainted with any other advanced technology besides solar energy. This is particularly convenient for Malaysia, as the country is situated in the equatorial zone, which provides it with a significant amount of sunlight all year round. This geographical advantage makes solar energy an especially viable and abundant renewable energy source for the country. Besides, the equatorial zone receives an abundance of sunlight, which presents a great opportunity for the solar industry to thrive. By investing in solar energy infrastructure such as solar farms and distributed solar installations, we can boost economic growth, create jobs, and attract investment in the renewable energy sector.

## 6 Conclusion

Malaysia is moving towards empowering renewable energy sources to generate electricity to deal with the worsening energy crisis. We need to stop importing coal to avoid the problem of inflation immediately. Furthermore, Malaysia is rich in renewable energy sources such as solar, biomass, biogas, and small hydropower. Thus, the country will suffer a massive loss if it does not fully utilize the renewable energy sources. One of the government's efforts to empower renewable energy sources as an alternative source in Malaysia is by introducing the Renewable Energy Fund (RE FUND) in 2011 until now. The manufacturing sectors can help achieve the government's targets through the RE FUND through the economic valuation of renewable energy sources. Since this sector uses the highest amount of electricity in Malaysia, investment to encourage the use of renewable energy sources is necessary. Besides, enabling renewable energy sources can also help achieve the Goal 7 in the Sustainable Development Goal (SDG), where investment in renewable energy sources can guarantee that all individuals obtain affordable, reliable, sustainable, and modern electricity by 2030. Based on the SDG Goal 7, transitioning from non-renewable to renewable energy sources reduces greenhouse emissions and promotes sustainable energy systems. Renewable energy technologies are commonly linked to high energy efficiency. Solar panels and wind turbines, for instance, can convert sunlight and wind into electricity with minimal harm to the environment. Investing in these technologies is in line to enhance overall energy efficiency.

This study chose the manufacturing sector as it consumes the highest amount of electricity in Malaysia compared to other sectors. Therefore, consumers who use more electricity need to contribute more to the RE FUND. This is in line with the approach where polluters have to pay more, where the party that pollutes more will pay more to the RE FUND. The implication of the RE FUND mechanism is a broader acceptance from consumers to take steps to reduce their electricity consumption through energy efficiency. This study found that the manufacturing sector chose the ‘project location’ attribute as their preference for investment in renewable energy sources. The respondents prefer that renewable energy project plants are located farther away from the factory. However, the limitation of this study is, that it does not provide a detailed distance between the project plants and factory locations. Therefore, it is imperative for future studies to establish a comprehensive measurement of the distance (in kilometers) between the project and the factory locations.

The research conducted on renewable energy is in accordance with Malaysia's national objectives, which are stated in the Malaysia Renewable Energy Roadmap (MyRER). The MyRER has been developed to aid Malaysia in achieving its vision of attaining a 31% share of renewable energy in the national installed capacity mix by 2025. Besides, the Twelfth Malaysia Plan, covering the period of 2021–2025, emphasizes the goal of achieving net-zero greenhouse gas emissions by 2050. The study's findings can provide helpful recommendations for renewable energy policy in Malaysia. For instance, policymakers can use the outcomes to develop relevant policies for renewable energy providers such as the Sustainable Energy Development Authority (SEDA) and the Energy Commission (EC), as well as the manufacturing sector. To achieve long-term benefits, policy changes are necessary. The findings of this study can serve as a guide for comprehensively restructuring the industry and regulatory policies to align with current needs and market demands. Investing less in renewable energy funds can restrict the ability of renewable energy companies to enhance their service quality and cover their capital expenses. Therefore, it is crucial to determine how much manufacturing sectors are willing to pay for renewable energy services for a particular level of service. This will help in understanding the manufacturing sectors' willingness to pay for renewable energy services in Johor, Malaysia. This study is also devoted to the development of empirical literature on the study of manufacturers' preferences and willingness to pay pertaining to renewable energy issues. This study also contributes to the literature on estimating economic benefits from improved renewable energy services by using the CE method, specifically in the manufacturing sectors in a developing country, Malaysia.

## Ethics declarations

All participants provided informed consent to participate in the study.

## Data availability statement

The data that support the findings of this study are available from the corresponding author, upon reasonable request.

## CRediT authorship contribution statement

**Siti Noradiah Amar:** Writing – original draft, Investigation, Formal analysis, Data curation, Conceptualization. **Mahirah Kamaludin:** Supervision, Software, Methodology, Investigation, Funding acquisition, Data curation, Conceptualization. **A. A. Azlina:** Writing – review & editing, Validation, Software, Resources, Methodology, Conceptualization. **Muhammad Rias K V Zainuddin:** Writing – review & editing, Visualization, Supervision, Resources, Conceptualization. **Khairul Izzuddin Sulaiman:** Validation, Resources, Data curation.

## Declaration of competing interest

This work was supported by the Fundamental Research Grant Scheme (FRGS/1/2018/SS08/UMT/02/1). The authors would like to thank the Ministry of Higher Education, Malaysia for the financial support.
