# Supplementary material for: Would manufacturing go for renewable energy? Manufacturers' preference towards sustainability
Source: Heliyon. 2024 Mar 16;10(6):e27981. doi: 10.1016/j.heliyon.2024.e27981 (PMC10958430; doi:10.1016/j.heliyon.2024.e27981)
Supplement: Multimedia component 1 [file mmc1.pdf]

## PART 1: CHOICE EXPERIMENT

The following is a summary of the attributes and attribute levels for the willingness of the manufacturing sector in Johor, Malaysia, to invest in renewable energy sources.

| Attributes | Attribute levels | Infographic | Descriptions |
|---|---|---|---|
| Types of renewable energy | **Level 1: Solar Photovoltaic**<br>Level 2: Biomass<br>Level 3: Biogas<br>Level 4: Small hydropower | 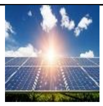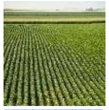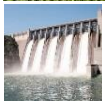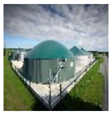 | The types of renewable energy sources responsible for supporting the government target to achieve 31% or 12.9GW of renewable energy installed capacity by 2025 and 40% or 18.0%GW of renewable energy installed capacity by 2035. |
| Project location | **Level 1: Unchanged**<br>Level 2: Near<br>Level 3: Far | 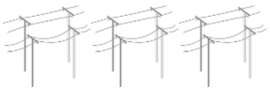 | The distance between the project location and the manufacturing sectors. |
| Annual reduction in GHG emission | **Level 1: 29.4%**<br>Level 2: 36.7%<br>Level 3: 45.0% | 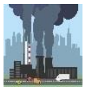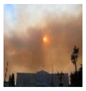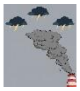 | The impact of renewable energy on greenhouse gas annual reduction. |
| Renewable energy fund | **Level 1: 1.6%**<br>Level 2: 2.2%<br>Level 3: 2.8% | 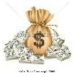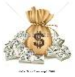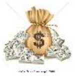 | Additional charges are implied to all manufacturing sectors except for factories that use electricity below 300kWh or RM77 per month. |

This study provides several choice sets of the willingness of the manufacturing sector in Johor, Malaysia, to invest in renewable energy sources. This study aims to find out which option will be a **PRIORITY** in the manufacturing sector for renewable energy sources.

**Which one is your choice?**
Please select ONE (1) of the THREE (3) alternatives provided based on preference criteria. Select the THIRD box if the alternatives do not meet your preference criteria.

**Notes:**

- Keep in mind if the alternative criteria are getting better, it requires the respondents to pay at a higher price rate.
- Keep in mind choosing to increase the monthly minimum charge will AFFECT THE MANUFACTURER'S MONTHLY EXPENDITURE COMMITMENT.

## CHOICE SETS

**Option 1:** If you are required to make a choice regarding electricity generation alternatives in your firm, but there are only alternatives A, B, & C, which option is your preferred choice? (Choose ONE only)*

| | **Alternative A** | **Alternative B** | **Alternative C** |
|---|---|---|---|
| Types of renewable energy | 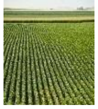 Biomass | 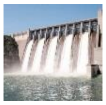 Small hydropower | There is no alternative that we can choose from |
| Project location | 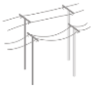 Far | 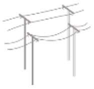 Unchanged | |
| Annual reduction GHG emission | 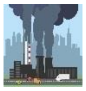 45.0% | 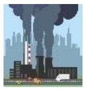 36.7% | |
| Increase in renewable energy fund | 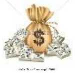 1.6% | 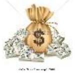 2.2% | |
| **Your choice?** | **[A]** | **[B]** | **[C]** |

**Option 2:** If you are required to make a choice regarding electricity generation alternatives in your firm, but there are only alternatives A, B, & C, which option is your preferred choice? (Choose ONE only)*

| | **Alternative A** | **Alternative B** | **Alternative C** |
|---|---|---|---|
| Types of renewable energy | 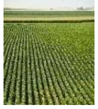 Biomass | 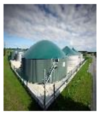 Biogas | There is no alternative that we can choose from |
| Project location | 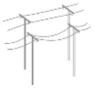 Far | 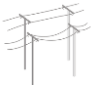 Unchanged | |
| Annual reduction GHG emission | 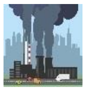 45.0% | 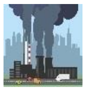 29.4% | |
| Increase in renewable energy fund | 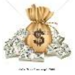 2.2% | 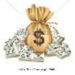 1.6% | |
| **Your choice?** | **[A]** | **[B]** | **[C]** |

**Option 3:** If you are required to make a choice regarding electricity generation alternatives in your firm, but there are only alternatives A, B, & C, which option is your preferred choice? (Choose ONE only)*

| | **Alternative A** | **Alternative B** | **Alternative C** |
|---|---|---|---|
| Types of renewable energy | 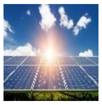 Solar | 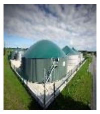 Biogas | |
| Project location | 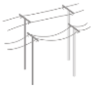 Near | 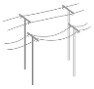 Unchanged | There is no alternative that we can choose from |
| Annual reduction GHG emission | 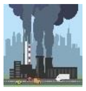 36.7% | 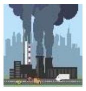 45.0% | |
| Increase in renewable energy fund | 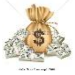 2.8% | 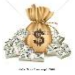 2.2% | |
| **Your choice?** | **[A]** | **[B]** | **[C]** |

**Option 4:** If you are required to make a choice regarding electricity generation alternatives in your firm, but there are only alternatives A, B, & C, which option is your preferred choice? (Choose ONE only)*

| | **Alternative A** | **Alternative B** | **Alternative C** |
|---|---|---|---|
| Types of renewable energy | 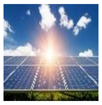 Solar | 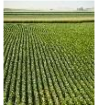 Biomass | |
| Project location | 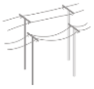 Unchanged | 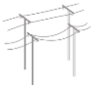 Far | There is no alternative that we can choose from |
| Annual reduction GHG emission | 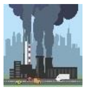 45.0% | 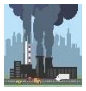 29.4% | |
| Increase in renewable energy fund | 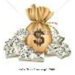 2.2% | 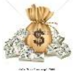 2.8% | |
| **Your choice?** | **[A]** | **[B]** | **[C]** |

**Option 5:** If you are required to make a choice regarding electricity generation alternatives in your firm, but there are only alternatives A, B, & C, which option is your preferred choice? (Choose ONE only)*

| | **Alternative A** | **Alternative B** | **Alternative C** |
|---|---|---|---|
| Types of renewable energy | 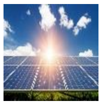 Solar | 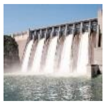 Small hydropower | There is no an alternative that we can choose from |
| Project location | 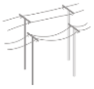 Far | 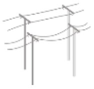 Unchanged | |
| Annual reduction GHG emission | 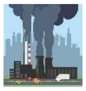 36.7% | 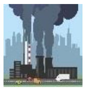 29.4% | |
| Increase in renewable energy fund | 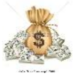 2.2% | 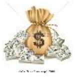 2.8% | |
| **Your choice?** | **[A]** | **[B]** | **[C]** |

**Option 6:** If you are required to make a choice regarding electricity generation alternatives in your firm, but there are only alternatives A, B, & C, which option is your preferred choice? (Choose ONE only)*

| | **Alternative A** | **Alternative B** | **Alternative C** |
|---|---|---|---|
| Types of renewable energy | 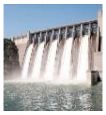 Small hydropower | 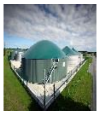 Biogas | There is no alternative that we can choose from |
| Project location | 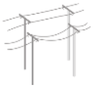 Unchanged | 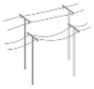 Near | |
| Annual reduction GHG emission | 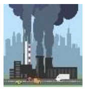 36.7% | 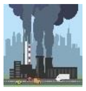 45.0% | |
| Increase in renewable energy fund | 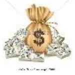 1.6% | 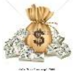 2.8% | |
| **Your choice?** | **[A]** | **[B]** | **[C]** |

**Option 7:** If you are required to make a choice regarding electricity generation alternatives in your firm, but there are only alternatives A, B, & C, which option is your preferred choice? (Choose ONE only)*

| | **Alternative A** | **Alternative B** | **Alternative C** |
|---|---|---|---|
| Types of renewable energy | 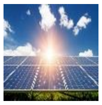 Solar | 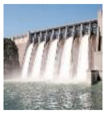 Small hydropower | There is no alternative that we can choose from |
| Project location | 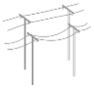 Far | 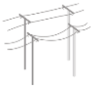 Near | |
| Annual reduction GHG emission | 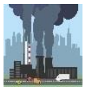 29.4% | 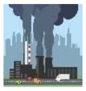 45.0% | |
| Increase in renewable energy fund | 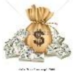 1.6% | 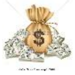 2.8% | |
| **Your choice?** | **[A]** | **[B]** | **[C]** |

**Option 8:** If you are required to make a choice regarding electricity generation alternatives in your firm, but there are only alternatives A, B, & C, which option is your preferred choice? (Choose ONE only)*

| | **Alternative A** | **Alternative B** | **Alternative C** |
|---|---|---|---|
| Types of renewable energy | 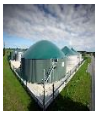 Biogas | 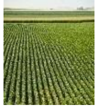 Biomass | There is no alternative that we can choose from |
| Project location | 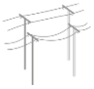 Far | 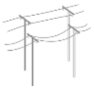 Near | |
| Annual reduction GHG emission | 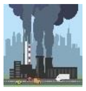 36.7% | 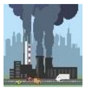 29.4% | |
| Increase in renewable energy fund | 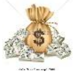 1.6% | 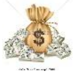 2.2% | |
| **Your choice?** | **[A]** | **[B]** | **[C]** |

Question 1. How confident are you in the choices that have been made?

| Strongly dissatisfied | Dissatisfied | Neutral | Satisfied | Strongly satisfied |
|---|---|---|---|---|
| ( ) | ( ) | ( ) | ( ) | ( ) |

Question 2. When making your decision, do you take into account the different sorts of renewable energy sources?
( ) Yes
( ) No

Question 3. When making your decision, do you take into account the project location?
( ) Yes
( ) No

Question 4. When making a decision, do you take into account the annual reduction in GHG emissions?
( ) Yes
( ) No

Question 5. When making a decision, do you take into account the increase in renewable energy funds?
( ) Yes
( ) No

**PART 4: RESPONDENT'S INFORMATION**

Question 9. Does this factory use electricity meters installed directly from Tenaga Nasional Berhad?
1. ( ) Ye – Continue answering Question 10.
2. ( ) No – The enumerator stops this survey and reports to the supervisor.

Question 10. Does this factory generate electricity using renewable energy sources?
1. ( ) Yes – Continue answering Question 11.
2. ( ) No – Continue answering Question 13.

Question 11. What type of renewable energy sources does the factory use to generate electricity?
1. Solar photovoltaic ( )
2. Biomass ( )
3. Biogas ( )
4. Small hydropower ( )
5. Others ( )

Question 12. What type of program does the factory use for the installation of renewable energy sources?
1. Feed-In-Tariff (FiT)
2. Net Energy Metering Program (NEM)
3. Large Scale Solar (LSS)
4. Self – Consumption (SELCO)
5. Renewable Energy Incentives

***If the factory produces electricity using renewable energy sources, disregard question 13***

Question 13. Is the factory interested in generating electricity by using renewable energy sources?
1. Yes ( )
2. No ( )

Question 14. Does the firm's electricity bill always exceed RM77 every month?
1. Yes ( )
2. No ( )

Question 15. What type of sub-sector is the production of this factory?

1. Electrical & electronics (    )
2. Petroleum, chemicals, rubber materials, and plastic materials (    )
3. Wood, furniture, paper, and printing (    )
4. Food, drink, and Tobacco (    )
5. Textiles, clothing, leather products, and shoes (    )
6. Non-metallic, mineral products, base metals, and fabricated metal products (    )
7. Transportation equipment and other manufacturing (    )

Question 16. Is your firm a registered firm in Malaysia?

( ) Yes
( ) No

Question 17. Is the firm aware of the existence of the Renewable Energy Fund?

( ) Yes
( ) No

**- Thank you for your kind cooperation -**
